# Salinity-Induced Changes of Photosynthetic Performance, Lawsone, VOCs, and Antioxidant Metabolism in *Lawsonia inermis* L.

**DOI:** 10.3390/plants9121797

**Published:** 2020-12-18

**Authors:** Basma Najar, Laura Pistelli, Ilaria Marchioni, Luisa Pistelli, Beatrice Muscatello, Marinella De Leo, Andrea Scartazza

**Affiliations:** 1 Department of Pharmacy, University of Pisa, 56124 Pisa, Italy; Basmanajar@hotmail.fr (B.N.); Luisa.pistelli@unipi.it (L.P.); Beatrice.muscatello@unipi.it (B.M.); Marinella.deleo@unipi.it (M.D.L.); 2 Department of Agriculture, Food and Environment (DAFE), University of Pisa, 56124 Pisa, Italy; i.marchioni@studenti.unipi.it; 3 Centre for Climate Change Impact (CIRSEC), University of Pisa, 56124 Pisa, Italy; 4 Research Institute on Terrestrial Ecosystems, Research National Council, 56124 Pisa, Italy; andrea.scartazza@cnr.it

**Keywords:** henna, abiotic stress, gas exchange parameters, photosynthesis, proline, antioxidant metabolites, dye compound

## Abstract

The present study aimed to elucidate the salinity influence on the bioactive metabolites of *Lawsonia inermis* L. (henna) plants. Young henna plants were cultivated under salinity stress with two NaCl concentrations (75 mM and 150 mM) in controlled environmental conditions and the leaves were investigated to check their adaptative responses. The modulation of photosynthetic performance to salinity stress was demonstrated by gas exchange and chlorophyll fluorescence parameters. The partial stomatal closure triggered an enhanced water-use efficiency, and a proline accumulation was observed, leading to an osmotic adjustment. The increased capacity to dissipate the excess excitation energy at photosystem II as heat was associated with changes in chlorophylls, anthocyanins, and carotenoids. The higher antioxidant activity at 150 mM salt level suggested its scavenger role on reactive oxygen species (ROS) dissipation and photoprotection. The reduced CO_2_ uptake and the higher metabolic costs necessary to sustain the henna tolerance mechanism against high NaCl concentration negatively affected lawsone production. Leaf volatile organic compounds (VOCs) showed changes in the amount and composition of VOCs with increasing salinity level. Overall, this study revealed efficient physiological and biochemical adaptations of henna leaves to salt stress despite an altered production of important economic metabolites such as lawsone.

## 1. Introduction

*Lawsonia inermis* L., commonly known as henna (Arabic), is a native plant of North Africa and Southwest Asia [1] that belongs to the Lythraceae family. Henna has been used cosmetically and medicinally for over 9000 years; its leaves contain different bioactive compounds, mainly alkaloids, phenols, steroids, and terpenes [1,2,3,4]. These compounds are known to have a wide range of biological properties such as antifungal, antibacterial, and antioxidant activities [5]. Henna is used worldwide as a cosmetic agent to stain hair, skin, and nails [2]. This use is due to the presence of lawsone (2-hydroxy-1,4-naphthoquinone), also known as hennotannic acid, a red-orange dye present in the leaves at a concentration of 1.0–1.4% *w/w* [5], with coloring and pharmacological activity. Lawsone is present only in the leaves and its concentration depends on climatic conditions. The cultivation of henna as a common hedge occurs in tropical and also arid areas characterized by very dry climatic conditions. In fact, henna plants perish at temperatures below 5 °C, and the reproduction phase is inhibited below 11 °C [6], while the dye compound is mainly produced between 35 and 45 °C.

Several studies have been carried out to underline the effect of some abiotic factors on germination, seedling growth, and morphological and physiological traits [7,8]. Drought stress is considered one of the main limiting factors of the henna cultivation that alters the growth, gas exchange parameters, and photosynthetic pigment contents proportionally with the intensity of water limitation [7,9]. *L. inermis* can overcome drought stress but not salinity [6] because it activates the reproductive phase when subjected to prolonged salt stress [10]. Nevertheless, the influence of salt stress on the lawsone production has not yet been determined. Bakkali and co-workers suggested that lawsone biosynthesis is affected by a complex control mechanism [11], and roots could play a role in the naphthoquinone biosynthesis. Therefore, the salinity treatment induced in the roots can contribute to changing the content of lawsone and other metabolites. It is well known that high levels of salinity induce generation of reactive oxygen species (ROS) that contribute to changes in membrane peroxidation [12]. In a recent paper [8], the positive effect of salicylic acid treatment emerged in terms of improving resistance to short-term salinity stress on young henna plants, with an activation of the antioxidant defense system through an increase in catalase activity.

Salt stress may also affect the essential oil (EO) production and composition in association with changes in enzymatic activity and alteration of intermediary products available during stress [13]. The influence of several environmental conditions on the emitted volatile organic compounds (VOCs) has been demonstrated on several medicinal and aromatic plants such as *Foeniculum vulgare, Majorana hortensis, Thymus vulgaris, Catharanthus roseus, Nigella sativa, Matricaria chamomilla, Salvia dolomitica,* and *Helicrysum petiolare* [13,14]. However, to the best of our knowledge, data concerning VOC production in *L. inermis* under salt stress are missing.

The aims of this paper were (i) to investigate the mechanism of stress tolerance to salt stress through the physiological and biochemical adaptations in henna plants grown for 20 days at increasing salinity levels, and (ii) to evaluate the effect of these adaptation mechanisms on plant growth, aroma compounds, and lawsone production.

## 2. Results

### 2.1. Morphological Parameters

The effects of salt treatments (75 mM and 150 mM NaCl) on the growth of henna plants were investigated at 0, 3, 10, and 20 days after treatment (DAT) (Table 1). The prolonged salt treatment pointed out the difference on plant growth correlated to the concentration of salt. Although the leaf number of henna was higher in control than in the treated plants, at 10 DAT, the discrepancy disappeared.

At 10 DAT, the 150 mM plants showed the lowest leaf number (13.87), while the control and 75 mM plants showed a higher number of leaves (14.62 and 16.62, respectively). At the end of treatment, the statistical difference was confirmed, and the number of leaves of the highest salt concentration was found to be lower than the other treatments.

The leaf area differed between and during the duration of treatments (Table 1). At 10 DAT, the highest salt concentration triggered a lower leaf area than that of the control. At the end of the treatment (20 DAT), the leaf area at 150 mM NaCl was about half that of the control, while 75 mM plants had values that were more linked to the control (2.744, 4.845, and 4.622 cm^2^).

At the end of treatment (20 DAT), the Relative Water Content (RWC) was determined, showing a lower percentage of water in 75 mM salt-stressed plants (71.63%) compared to the control (80.29%), followed by those at 150 mM (74.35%) (Table 1).

### 2.2. Effects of Salt Treatment on Chlorophyll Fluorescence Parameters and Leaf Gas Exchanges

**Figure 1:**
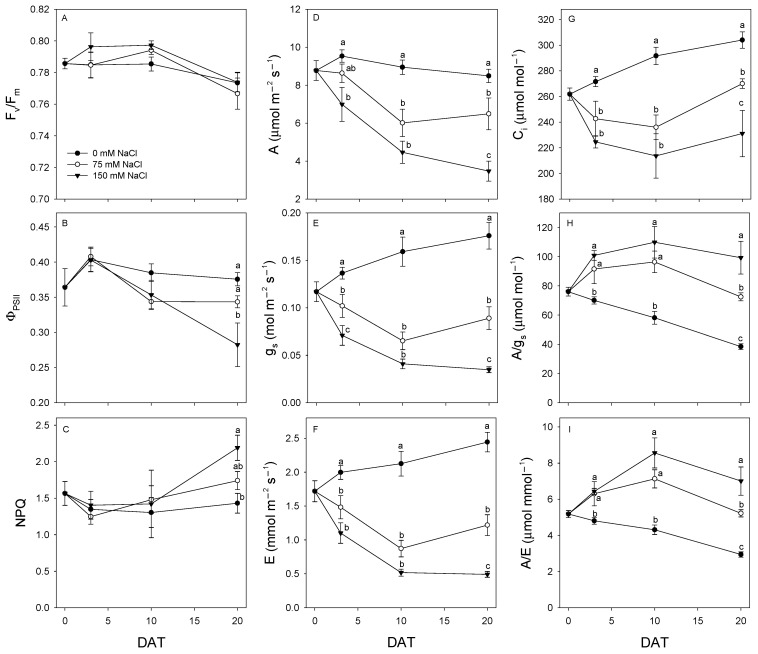
Variations of (**A**) potential efficiency of photosystem II (PSII) photochemistry (F_v_/F_m_), (**B**) actual photon yield of PSII photochemistry (Φ_PSII_), (**C**) non-photochemical quenching (NPQ), (**D**) CO_2_ assimilation rate, (**E**) stomatal conductance (g_s_), (**F**) transpiration rate, (**G**) intercellular CO_2_ concentration (C_i_), (**H**) intrinsic water-use efficiency (A/g_s_), and (**I**) instantaneous water-use efficiency (A/E) at 0, 3, 10, and 20 days after treatment (DAT) with 0, 75, and 150 mM NaCl. Values are means ± SE (*n* = 6–9). Different letters indicate significant differences among treatments (*p* ≤ 0.05, Fisher’s Least Significant Difference, LSD).

The effects of salt treatments (75 mM and 150 mM NaCl) on photosynthetic-related parameters were monitored by chlorophyll fluorescence and leaf gas exchange measurements at 0, 3, 10, and 20 days after treatment (DAT) (Figure 1). The analysis of the fluorescence of chlorophyll *a* showed significant difference in Φ_PSII_ and non-photochemical quenching (NPQ) only at 20 DAT of the highest salt treatment, while F_v_/F_m_ remained unchanged throughout the treatment period (Figure 1A–C). In detail, at 20 DAT, plants treated with 150 mM NaCl showed a significant reduction of Φ_PSII_ associated with an increase in NPQ, while plants treated with 75 mM NaCl did not show any significant changes of the fluorescence parameters with respect to control throughout the treatment period.

Figure 1D–I shows the variations of A, g_s_, E, C_i_, intrinsic water-use efficiency (WUE) (i.e., A/g_s_), and instantaneous WUE (i.e., A/E) during the treatment period. Plants treated with 150 mM NaCl showed a lower A compared to control only after 3 days of treatment, while those treated with 75 mM NaCl were not significantly affected by the treatment until 10 DAT. At 3 DAT, a decrease of g_s_ occurred at both the NaCl concentrations, although the stomatal closure was more marked in plants treated with the highest salt dose. The gas exchange parameters C_i_ and E showed a similar decrease in both salt treatments at 3 DAT, while A/gs and A/E significantly increased compared to the control. After 10 days of salt treatment, a similar decrease of A, g_s_, C_i_, and E was observed at both salt concentrations compared to the control, while A/g_s_ and A/E were significantly higher in salt-treated plants than in the control. At 20 DAT, a dose-dependent reduction of A, g_s_, C_i_, and E was observed in salt-treated plants compared to the control, with the lowest values for all these gas exchange parameters recorded in plants treated with the highest NaCl concentration. Conversely, A/g_s_ and A/E were maintained significantly higher in salt-treated plants than in the control, especially at the highest NaCl concentration.

### 2.3. Biochemical Analyses

Analyses of several metabolites were performed at 20 DAT (Table 2). The salinity produced different effects on photosynthetic pigments, which were measured per area of fresh leaves. The amount of chlorophyll *b* and *a* was significantly higher at the highest NaCl concentration (6.7 and 20.95 mg cm^−2^ per 150 mM, respectively) in comparison with the other plants (3.7 and 12.47 mg cm^−2^, respectively, in control vs. 5.22 and 15.68 mg cm^−2^, respectively, at 75 mM). Therefore, the total chlorophyll amount gradually increased with the salinity (16.17, 20.9, and 27.65 mg cm^−2^ for control, 75 mM, and 150 mM NaCl, respectively). However, the ratio of chlorophyll *a*/chlorophyll *b* showed a decrease in the salinity treatment. The total carotenoid content showed an increased concentration in the presence of the highest salt level, reaching a 1.5-fold value of the other salt level (5.2, 5.86, and 7.4 mg cm^−2^ for control, 75 mM, and 150 mM NaCl, respectively). Anthocyanin pigments followed a similar trend of carotenoids; the highest value (48.96 mg g^−1^ Fresh Weight, FW) was detected in 150 mM henna plants, while a lower concentration of salt did not produce a significant difference in comparison with the control (34.53 and 29.53 mg g^−1^ FW, respectively; Table 2). Anthocyanins are chemically ascribed as the main class flavonoids, and the total amount of these metabolites increased with the NaCl concentration, with a 1.5-fold higher amount in 150 mM leaves in comparison with the control (551.52 and 375.82 mg g^−1^ FW, respectively).

The salt stress is often associated with oxidative stress, and therefore the antioxidant activity was determined as radical scavenger activity of 2,2-diphenyl-1-picrylhydrazyl radical (DPPH) using the IC_50_ values. The DPPH assay was higher in the leaves of plants treated with 150 mM NaCl, which exhibited the highest value of fresh leaves reducing 50% of DPPH (16.07 mg mL^−1^), while the lower activity was detected in the control (24.51 mg mL^−1^), and the 75 mM NaCl showed a medium level of activity (19.72 mg mL^−1^). Using a different antioxidant test (ferric ion reducing antioxidant power (FRAP) assay), we found that the obtained values confirmed the increased antioxidant activity with the increase of salt concentration. Polyphenols are considered a class of metabolites involved in several stress conditions, but the influence of salt stress in *L. inermis* leaves did not include changes in their levels, since no significant difference was demonstrated (Table 2). The salinity showed an increase in proline content. Control leaves showed the lowest amount (70.16 mg g^−1^ FW), and the highest amount (statistically different) was detected in 150 mM (91.3 mg g^−1^ FW).

### 2.4. Lawsone Production

Results about lawsone extraction from plants subjected to control (0 NaCl) and saline stress (75 and 150 mM NaCl) are reported in Table 3. The extraction process was not highly selective, and thus other high polar molecules were also concurrently extracted. The yield of the control plant extraction was much higher than that obtained from the treated plant extractions. Lawsone was detected and quantified in all the samples by liquid chromatography (LC)–photo diode array (PDA)/ultraviolet (UV)–electrospray ionization (ESI)–tandem mass spectrometry (MS/MS) analyses. The presence of lawsone was confirmed in all the extracts, comparing its UV and mass spectra with those of a reference standard. UV spectra were characterized by three absorptions at 248, 289, and 333 nm. In the full mass spectrum, the deprotonated molecule [M−H]^−^ was detected at *m/z* 173, while the fragmentation pattern showed a diagnostic product ion at *m/z* 145 due to the neutral loss of a CO molecule [M–H–28]^−^. The lawsone amount (Table 3), expressed as milligram of compound in 1 g of fresh material, differed among the analyzed samples when compared to the control (5.90 mg g^−1^ FW). Notably, both saline treatments (75 and 150 mM) induced a significant decrease (about 77% and 80%, respectively) in term of lawsone amount.

### 2.5. VOC Evaluation

The relative percentage of volatile compounds in both normal and salt-stressed leaves of *L. inermis* analyzed by Gas Chromatography–Mass Spectrometry (GC–MS) is reported in Table 4. Almost the half of compounds were in common in all the samples (a total of 26). Each sample was separated from the others by some compounds whose number increased with the salinity concentration. These components represented 3% of the identified fraction in control plants, reached 7% in 75 mM, and overcame 32% in the samples treated with 150 mM NaCl. Salt stress induced the biosynthesis of 18 new compounds at 75 mM and 13 at 150 mM. These compounds belonged to various chemical classes. Aliphatic hydrocarbons (non-terpenes, NTs) were the major class in the control sample and in the sample stressed at 150 mM NaCl, accounting for 55.5% and 63.5%, respectively (Table 4).

The aromatic compounds (total terpenes) were the second chemical class, showing almost the same percentage in control (32.4%) and in 150 mM NaCl-stressed plants (30.1%). Plants treated with 75 mM NaCl evidenced a controversial behavior because the most important class of constituent was of the total terpenes (50.1%) followed by non-terpenes (38.6%). Of note was the drastic decrease in the apocarotenoid percentage with the increase of salinity. The application of NaCl conspicuously decreased the amount of this latter class, exclusively represented by (E)-geranylacetone, in amounts of 37% and 88% for 75 and 150 mM NaCl, respectively. This decrease also affected the alcohols (71% and 63%, respectively). The variation in monoterpene hydrocarbon content with the increase of salinity is also important to mention. Their amount increased at 75 mM (↑62%) and fell below the control value (↓62%) at 150 mM NaCl. The same trend was also observed in oxygenated monoterpenes (↑36% and ↓22%, respectively), total terpenes (↑55% and ↓7%, respectively), aldehydes (↑41% and ↓34%, respectively), and the sum of ethers and ketones (↑92% and ↓46%, respectively). On the contrary, the number of constituents increased from 32 in the control, to 47 in 75 mM, to 51 at 150 mM NaCl. Moreover, the highest NaCl concentration (150 mM) induced a huge increase of alkanes (1300%) and esters (226%), together with the percentage of sesquiterpene compounds (194%).

The control specimens were characterized by the highest relative abundance of (*E*)-3-hexen-1-ol (27.0%), whose percentage decreased by 69.3% at the highest concentration of NaCl, where (*Z*)-3-hexenyl isovalerate became the main constituent (20.9%). Compared to the control, relative content in (*Z*)-β-ocimene lowered at 47.8% in 75 mM NaCl and then disappeared at 150 mM NaCl, and the same occurred for γ-terpinene, *n*-eicosane, and 3-ethyl-1-hexanol. The application of 75 mM NaCl did not notably change the linalool content. However, the level of 150 mM NaCl caused a significantly negative effect on its content (a decrease of 40.7%). In contrast, NaCl stress enhanced the amount of τ-cadinol and methyl 3,5-di-tert-butyl-4-hydroxybenzoate.

Among the identified compounds of the leaves, nine evoked common green leaf volatiles (GLVs); in fact (*E*)-3-hexen-l-ol, 1-hexanol, 3-ethyl-1-hexanol, nonanal, (*Z*)-3-hexenyl acetate, hexyl acetate, (*Z*)-3-hexenyl 3-methylbutanoate, hexyl 3-methylbutanoate, and (*Z*)-3-hexenyl isovalerate were included. As shown in the Table 4, treatment with NaCl promoted a slight decrease in the total percentage of the identified GLVs, even though their number increased when compared with the control.

**Figure 2:**
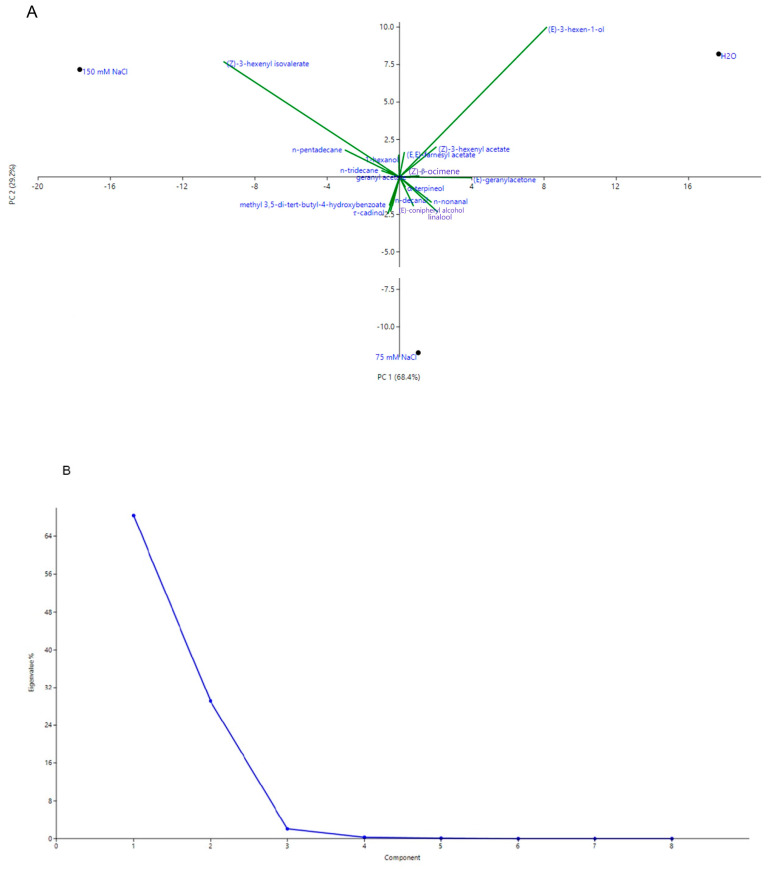
Scatter plot (**A**) and Scree plot (**B**) of the principal component analysis (PCA) of the volatile organic compounds (VOCs) at different gradient of salinity.

Principal component analysis (PCA), where the first two axes added up to 97.0% of the total variability (Figure 2A,B), showed two first macro groups, one with positive loading on PC1 and the other one with a negative loading on the same axes. Control plants and those treated with 75 mM NaCl were plotted in the area of positive scoring on PC1, where they were divided into two bottom quadrants—control samples were plotted on the bottom right quadrant (positive loading on PC2), whilst samples exposed to medium salt stress (75 mM) were plotted on the bottom left quadrant (negative loading on PC2). However, the only sample that scored a negative loading on PC1 was that treated with 150 mM NaCl—it was on the left quadrant, thus with a positive score on PC2.

This position was due to their contents in (*Z*)-3-hexenyl isovalerate, which was the only variable whose loading was negative along PC1 and positive on PC2. It is important to notice that all the variables were concentrated around the origin of the axes, and only (*E*)-3-hexen-1-ol and (*Z*)-3-hexenyl isovalerate were more dispersed.

**Figure 3:**
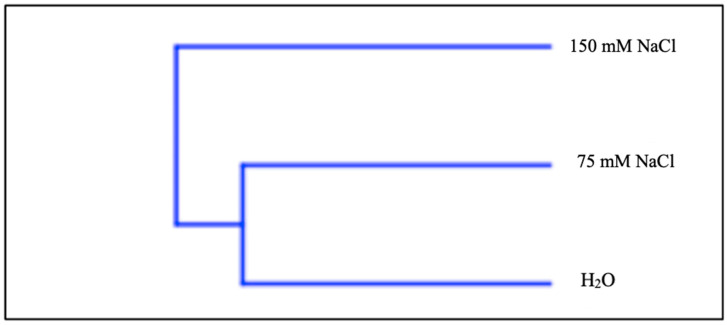
Dendrogram of the hierarchical cluster analysis (HCA) of the VOCs at different gradients of salinity.

Hierarchical cluster analysis HCA (Figure 3), performed with the total data of the volatile composition, fit perfectly with the PCA results (Figure 2). In fact, HCA showed two different groups—A and B. Group A included only the sample at 150 mM NaCl, while group B gathered together the control and 75 mM NaCl.

The one-way PERMANOVA performed on the VOC compounds revealed significant differences among the salinity gradient (*p* = 0.0003) (Table 5).

According the SIMPER analysis, 13 compounds were responsible for more than 95% of this dissimilarity (Table 6), with (*Z*)-3-hexenyl isovalerate (39.86%) and (*E*)-3-hexen-1-ol (35.99%) being the major contributors, together covering more than 75% of the difference.

## 3. Discussion

### 3.1. Effect of Salt Treatment on Growth of Plants

Salinity influences the growth and development of plants by limiting leaf expansion [12] and also fruit production such as in tomato [15]. In the present work, young henna plants showed a dose-dependent reduction of growth under a short-term period (up to 20 days) of salt treatment. In particular, our data showed the salt tolerance of henna plants at medium concentration of NaCl (75 mM), which did not show any statistical difference in leaf number and leaf area compared to control plants. Conversely, an inhibition of growth parameters occurred at the highest salt level (150 mM) after only 20 days of treatment. A similar reduction of leaf number and leaf area was observed in henna plants subjected to drought stress [7] and to long-term salinity treatment [10].

The reduced leaf area is considered an avoidance mechanism to minimize water loss by transpiration, together with the stomatal closure, as has already been reported for the Mediterranean plant *Rosmarinus officinalis* [16]. This species, like other tolerant plants, operates an osmotic adjustment to maintain its water status under salt stress, as supported by the slight reduction in RWC [16].

### 3.2. Effects of Salt Treatment on Photosynthetic Parameters

Henna plants were able to respond to salinity by maintaining unaltered values of the potential efficiency of photosystem II (PSII) photochemistry (close to 0.8) throughout the treatment period independently of the NaCl dose, suggesting the absence of PSII damages [17,18]. However, at severe salt stress (150 mM NaCl), a decrease of the actual photon yield of PSII photochemistry was observed after 20 days of salt treatment associated with an increase of NPQ, which furnishes an estimation of thermal energy dissipation capacity [19]. The effect of salinity stress on non-photochemical quenching parameters is dependent on plants and cultivar, increasing more substantially in the salt-tolerant than in the salt-sensitive plants [20]. These data suggest that henna plants were able to counteract the increase of excess excitation energy at PSII due to the reduced electron transport rate by increasing heat dissipation; this contributes to avoid photoinhibition and photodamage of photosynthetic apparatus under high salinity level [21]. Accordingly, henna plants showed a decrease of both A and C_i_ compared to control plants, supporting the hypothesis that the salt-dependent decrease in photosynthetic CO_2_ uptake was mainly due to stomatal factors, rather than to PSII activity reduction or metabolic impairment [22,23]. Indeed, henna plants showed a dose-dependent decrease of g_s_ after salt treatment, in agreement with previous findings, suggesting that regulation of stomatal conductance plays an important role in the adaptation to high salinity in this species [10] and in other semi-arid adapted plants [15,24]. The partial stomatal closure reduced the leaf transpiration in henna plants, contributing to maintaining the leaf turgor potential and to reducing the influx of salt into the shoots [12]. The decrease of A in henna plants was not as severe as the decrease in g_s_ and E, leading to an increase in both intrinsic and instantaneous WUE, especially under severe salt stress [10]. As a consequence, henna plants showed only a slight reduction of RWC under both salt treatments, similar to that observed in wheat, known as medium tolerant species [25]. Hence, our data pointed out a crucial role of heat dissipation of excess energy, stomatal control, and increase in WUE in the adaptation of henna plants to high salinity levels.

### 3.3. Effects of Salt Treatment on Lawsone Production and Stress-Related Metabolic Compounds

Henna plant is widely cultivated for the production of the lawsone, a dye used both as a cosmetic and a pharmacological agent [5]. Lawsone production is enhanced by high temperature and high light intensity [6]. Many reports have been conducted to analyze the growth condition of henna in order to achieve information on its ability to tolerate drought and salinity [7,8,10]. However, these authors excluded the examination of the influence of the stress for the production of lawsone. In the present work, lawsone content was determined for the first time during salinity exposition for 3 weeks. The amount found in the leaves of henna treated with different NaCl concentrations revealed that salinity led to a decreased production, associated with the decrease in photosynthetic activity. Hence, our data highlighted that the reduction in lawsone production was related to the reduced CO_2_ uptake under salinity treatment, essentially due to the partial stomatal closure.

Salt stress can affect the photosynthetic apparatus, leading to salt accumulation in young leaves and loss of photosynthetic pigments [26]. However, plants tolerant to NaCl respond to salinity by maintaining or increasing their chlorophyll content, suggesting that this parameter can be considered a biochemical marker of salt tolerance in plants [20]. Hence, the increase of total chlorophylls in henna plants with increasing NaCl concentration indicated a high resilience of this species to salt stress, protecting the photosynthetic apparatus from irreversible damages. In particular, the observed changes in photosynthetic pigments could be involved in the optimization of light capture and dissipation of excess energy under salt stress conditions [22,27,28]. Likewise, the NPQ increased in 150 mM NaCl-treated plants, indicating the activation of the photoprotection process. This statement is supported by the increasing amount of carotenoids. Indeed, carotenoids operate an important photoprotective role by dissipating excess excitation energy at PSII as heat through the so-called xanthophyll cycle and can play a crucial role in protecting photosynthetic apparatus from photoinhibitory damage under high salinity level [21]. Moreover, under stress conditions, carotenoids also act as scavenging agents against ROS, protecting the photosynthetic apparatus from oxidative stress [29]. However, salinity tolerance is often attributed to an osmotic adjustment and the stomatal control linked to the accumulation of osmolytes or compatible solutes [12]. The closure of stomata in salt stress is well documented, and proline thus represents most important osmoprotectant agent, for which concentration is enhanced under salinity conditions [12]; this is in agreement with the data reported in henna leaves, where proline was found to be significantly greater in 150 mM leaves. Moreover, proline can have antioxidant properties, protecting the structure of macromolecules against the dehydration process.

Under salinity conditions, one of the most common responses of plants is the accelerated generation of ROS produced in the photosynthetic process, and thus an efficient antioxidant system is needed to counteract the oxidative burst associated with ROS production [20]. Therefore, the antioxidant compounds as polyphenols, anthocyanins, and flavonoids were monitored during salt treatment in *L. inermis*. In agreement with the antioxidant activity (DPPH assay), anthocyanins and flavonoids were more concentrated in the highest salt treatment, while medium stress did not influence these molecules. The ability to modulate the mechanism of production of antioxidant molecules with the strength of salinity is already known in several plants [30], and *L. inermis* is considered a medium tolerant species to salt stress [8,10].

### 3.4. Emission of Leaf VOCs in Henna Plants Subjected to Salt Stress

Plants produce volatile compounds to cope with environmental stress and avoid damage [31]. VOC release under NaCl stress is helpful for plants, driving the maintenance of stomatal conductance and photosynthesis [32]. The control leaves of *L. inermis* showed similarity with the EO composition of Tunisian henna leaves, which has already been published [33]. The presence of β-ionone, a typical compound of different henna powder and leaves, was not revealed in the VOCs of this work [34,35]. Moreover, no information is available regarding the salt effect on VOCs of *L. inermis*.

The leaves were rich in both oxygenated monoterpenes and alcohols, but they were negatively affected by salt stress. The same behavior was also noted in monoterpene hydrocarbons, aldehydes, and the sum of ethers and ketones. Conversely, total sesquiterpenes were enhanced at high salt concentration. Several terpenoids, such as (*E*)-β-ocimene, linalool, b-caryophyllene, and (*E*)-β-farnesene, are characteristic stress compounds in many plant species [36]. Except for the two first compounds, which were already present in the VOCs of control plants, the percentage of the remaining two was increased by the salinity stress.

Other volatile compounds belonging to esters, alkanes, and alcohols are recognized as Green Leaf Volatiles (GLVs) and are defined as a class of six-carbon (C6) compounds that play a role in plant defense against insect herbivore attack [37]. Our results revealed the presence of these compounds even in the control plants, although total alkanes and, especially, esters showed a growing trend with an increase in the salinity level, gradually becoming one of the dominant classes in salt-treated plants at 150 mM NaCl, while the monoterpene hydrocarbons decreased. In fact, GLVs were responsible for segregating the control samples from those treated with salt, as shown in PCA analysis. A reduction of monoterpenes associated with an enhanced biosynthesis and accumulation of esters has been previously observed in *Schizonepeta tenuifolia* as salinity increased [38]. Moreover, it has been shown that the treatment with GLV esters induced stomatal closure in several plant species belonging to Solanaceae, Leguminosae, Brassicaceae, Citrus, and Gramineae [39]. Tomescu and co-workers [40] showed a drastic effect of salinity on the emission of some GLVs in *L. esculentum leaves* as a product of lipoxygenase pathway, as well as the increased emission of all terpenes in proportion to the salt concentration. It is likely that the emission of such compounds could be elicited as in response to other abiotic stresses such as high light, ozone, and high temperature [31]. It is interesting to note the significant decrease of apocarotenoids induced by salinity stress. These compounds are ubiquitous carotenoid derivatives and include volatile aromatic compounds as well as the phytohormones abscisic acid and strigolactones, which could be synthesized during the salt stress in *L. inermis.*

Overall, our data highlighted significant changes in VOC production in leaves of *L. inermis* subjected to salt stress, with an increase of specific volatile compounds such as some terpenoids, alkanes, and esters. However, further studies will need to be carried out in order to support the involvement of these compounds in the salt tolerance mechanisms of *L. inermis* and to unravel their interaction with the observed physiological and biochemical adaptations.

## 4. Materials and Methods

### 4.1. Plant Material and Growing Conditions

Seeds of *Lawsonia inermis* L. were bought from a market in Gabès (South Tunisia). Seeds were sterilized by immersion in 35% sodium hypochlorite (NaClO) for 5 min, then rinsed in water and soaked in 70% ethanol for 10 min. After rinsing 3 times with distilled water, the seeds were placed in Petri dishes at 4 °C for 3 days. After this treatment, the seeds were transferred in rockwool plug trays (Grodan 105 Pro Plug) for hydroponic cultivation using Hoagland solution as a nutrient. The germinated plantlets were maintained in a growth chamber at 25 ± 1 °C, 60 ± 5% of relative humidity (RH), and under 16/8 h photoperiod provided by cool white fluorescent tubes (Philips TLM 40 W/33RS) with 100 μmol m^−2^ s^−1^ photosynthetic active radiation (PAR). After 1 month of hydroponic culture and growing, uniformly sized plantlets were transferred into pots (10 pots × treatment, 1 plant × pot) containing 0.45 L of soil composed of 45% clay, 45% sand, and 10% silt under 16 h photoperiod provided by cool white fluorescent tubes (Philips TLM 40 W/33RS) with 500 μmol m^−2^ s^−1^ PAR. The plants (approximately 10 cm long) were randomly divided into 3 groups (10 plants per group) for each salt stress level. Treatment was applied every other day with 50 mL of 0 mM (control), 75 mM, and 150 mM NaCl for 20 days and once a week with the Hoagland solution. At the end of the trials, leaves were homogenously harvested and used fresh or stored at −80 °C, depending on the morphological, physiological, and biochemical analyses.

### 4.2. Morphological Parameters

At 0, 3, 10, and 20 days after treatment, the number of leaves was recorded in each pot. The relative leaf area was measured for each plant at 10 and 20 days with a digital planimeter. Relative water content (RWC) was determined using fresh leaf discs of 1 cm^2^. After weighting, the leaf discs were immersed in deionized water for 24 h and excess water was wiped with tissue paper. Full Turgor leaf weights were recorded, and the dry masses were measured after drying at 60 °C for 48 h. The RWC was calculated as: RWC = [(Fresh weight − dry weight)/(Turgor weight − dry weight)] ×100.

### 4.3. Gas Exchange and Chlorophyll Fluorescence Measurements

Gas exchange and chlorophyll fluorescence measurements were performed on fully expanded and exposed leaves of *L. inermis* at 0, 3, 10, and 20 days after treatment with 0, 75, and 150 mM NaCl. Measurements were carried out using a portable infrared gas analyzer (LI-6400-40, LI-COR Inc., Lincoln, NE, USA) equipped with the leaf chamber fluorometer. Instantaneous measurements of steady-state CO_2_ assimilation rate (A_,_ µmol CO_2_ m^−2^ s^−1^), stomatal conductance (g_s_, mol H_2_O m^−2^ s^−1^), intercellular CO_2_ concentration (C_i_, µmol CO_2_ mol^−1^), transpiration rate (E, mmol H_2_O m^−2^ s^−1^), actual photon yield of PSII photochemistry (Φ_PSII_), Stern–Volmer non-photochemical quenching (NPQ), and the potential efficiency of PSII photochemistry (F_v_/F_m_) were performed between 09:00 and 11:00 a.m. under growing Photosynthetic Photon Flux Density (PPFD, 500 μmol photons m^−2^ s^−1^), CO_2_ concentration of 400 μmol mol^−1^, and leaf temperature of 25 °C, as reported in [41]. Intrinsic and instantaneous water-use efficiency (WUE) values were determined as A/g_s_ and A/E ratio, respectively. Measurements of F_v_/F_m_ were performed after at least 30 min of acclimation in the dark. The actual photon yield of PSII photochemistry was determined as Φ_PSII_ = (F_m_’ − F_s_)/F_m_’ [42] at steady state, where F_m_’ is the maximum fluorescence yield with all PSII reaction centers in the reduced state obtained by superimposing a saturating light flash during exposition to actinic light, with F_s_ being the fluorescence at the actual state of PSII reaction centers during actinic illumination. Non-photochemical quenching was determined according to the Stern–Volmer equation as NPQ = (F_m_/F_m_’) − 1, where F_m_ is the maximum fluorescence yield in the dark, as in [43].

### 4.4. Biochemical Analyses

Fresh leaves (0.1 g), homogenously sampled (20 leaves × treatment) from the mid-lamina area of the intervene zone, were used for determination of pigments (chlorophylls and carotenoids), total polyphenols, total flavonoids, and 2,2-diphenyl-1-picrylhydrazyl radical (DPPH) antiradical activity, according to the published protocols [44] using a UV–VIS spectrophotometer (SHIMADZU UV-1800). The extract was also tested with ferric ion reducing antioxidant power (FRAP) antioxidant assay to confirm the antioxidant activity [45]. Anthocyanins were extracted from 0.1 g of fresh leaves in ethanol/HCl (99/1, *v/v*) and used to read the absorbance at 535 nm [46]. The total anthocyanin content was expressed as milligrams of malvin chloride equivalents (ME) per gram of fresh weight. The data presented are the mean of 3 independent replicates of the homogenously pooled sample. Proline (Pro) content was determined following Bates [47], after extraction with sulfosalicylic acid (3%, *v/v*). Spectrophotometric determinations were performed at 520 nm, using toluene as a blank.

### 4.5. Lawsone Determination

Lawsone was extracted using a method reported by Bakkali [11], with some modifications. Fresh aerial parts (1.0 g) of *L. inermis* related to control (0 NaCl) and salt treatment (75 and 150 mM NaCl) were dried at 30 °C and powdered, and therein extracted with 25 mL of EtOH-H_2_O 45% (*v/v*) for 20 h under stirring (90 rpm). Each obtained extract solution was filtered and acidified with 50 μL of acetic acid, then diluted with 25 mL of EtOH-H_2_O 45% (*v/v*) and extracted twice in a separating funnel with chloroform (50 mL). The combined chloroform solutions were dried by Rotavapor (BUCHI, ITALY) at 39 °C, then dissolved in methanol at a final concentration of 2.0 mg mL^−1^ and centrifuged (for 5 min at 1145× *g*) for injection in a high-performance liquid chromatography (HPLC)–photo diode array (PDA) –electrospray ionization (ESI) –mass spectrometry (MS) system.

Quantitative analysis of lawsone was performed using HPLC–PDA/UV–ESI–MS/MS equipment composed by Surveyor LC pump and autosampler coupled with a Surveyor PDA/UV–VIS detector and an LCQ Advantage ion trap ESI–MS (THERMOFINNIGAN, San Jose, CA, USA). All henna samples and standard lawsone were injected (20 μL injection volume) on a Synergi Fusion-RP column, 4.6 × 150 mm, 4 μm particle size (PHENOMENEX, Bologna, ITALY), eluting with a mobile phase consisting of methanol (solvent A) and formic acid in water 0.1% *v/v* (solvent B) using the following gradients: 0–15 min 48% A isocratic mode, 15–17 min 48–100% A, 17–27 min 100% A isocratic mode, 27–29 min 100–48% A, 29–39 48% A isocratic mode. The elution was performed at a flow rate of 1.0 mL min^−1^, using a splitting system of 2:8 to MS (200 μL min^−1^) and PDA (800 μL min^−1^) detectors, respectively. MS experiments were performed in a positive ionization mode using ionization parameters previously reported [48] and by applying normalized collision energy 35.0% in the MS/MS experiments. N_2_ was used both as sheath and auxiliary gas. PDA/UV–VIS experiments were registered at 248, 289, and 333 nm (absorptions observed for lawsone) as preferential channels. MS data were analyzed by Xcalibur 3.1 software.

The lawsone amount in all henna samples was obtained using pure lawsone as external standard in a concentration range of 125–1000 μg mL^−1^. Lawsone standard solutions were prepared at 4 concentrations (1000, 500, 250, and 125 μg mL^−1^) and injected in triplicate into the LC–PDA/MS system. Area obtained from the integration of peaks recorded at 289 nm was used for constructing the calibration curve. A linear simple correlation was used to determine the relation between variables. For the linear regression of the standard, *R*^2^ was 0.9632. The lawsone amount was expressed as mg g^−1^ of fresh material. Data were obtained by using a GraphPad Software Prism 6.0.

### 4.6. VOC Analysis

The analyses of spontaneous emission (VOCs) were performed by HeadSpace–Solid Phase MicroExtraction (HS–SPME) using a polydimethylsiloxane (PDMS)-coated fiber. Homogenous samples of fresh leaves (2 g) were introduced into a glass conical flask (20 mL) and left to equilibrate for 30 min. The fiber was exposed to the headspace for 20 min at room temperature; the fiber was then reinserted into the needle and transferred to the injector of the GC–MS systems where it was desorbed. GC–MS analysis was performed by a Varian CP-3800 gas chromatograph equipped with a DB 5 capillary column (30 m × 0.25 mm; coating thickness: 0.25 μm) and a Varian Saturn 2000 ion trap mass detector. Analytical conditions and constituent identification are cited in a previous work [49].

### 4.7. Statistical Analysis

Data were submitted to different multivariate analyses. A matrix of variance–covariance was used for the measurement of eigenvalues and eigenvectors in PCA analysis, wherein the plot was performed by selecting the 2 highest principal components (PCs). This analysis, which concerned only compounds with a significant difference between the treatments and which showed a percentage greater than 2%, aimed at reducing the dimensionality of the multivariate data of the matrices whilst preserving most of the variance. The hierarchical cluster analysis (HCA) was performed using Ward’s method with squared Euclidian distances as a measure of similarity to individuate possible clusters of samples in the dataset. Statistically significant differences induced by salinity on VOCs were assessed with the one-way PERMANOVA with Euclidean index similarity. The percentage contribution of each compound to the observed dissimilarity was assessed through the similarity percentage analysis (SIMPER, Euclidean distance). For each compound, the difference between different salinity gradient tested with one-way ANOVA using Least Significance Difference (LSD) test for the post hoc analysis. The ANOVA analysis was performed with SPSS software (version 21), while all other analyses were performed with Past software (version 3). Morphological and biochemical data, gas exchange parameters, and chlorophyll fluorescence were statistically analyzed by ANOVA, followed by Fisher’s probable least-squares difference test with cut-off significance at *p* ≤ 0.05 (letters).

### 4.8. Chemicals

Ethanol, chloroform, acetic acid, HPLC-grade formic acid, and methanol were purchased from VWR (Milan, Italy). HPLC-grade water (18 mΩ) was obtained by a Mill-Q purification system (Millipore Corp., Burlington, MA, USA). Standard lawsone (purity 97%) was purchased from Sigma-Aldrich (Milan, Italy).

## 5. Conclusions

*L. inermis* subjected to increasing NaCl concentrations showed an alteration of proline, photosynthetic pigments, antioxidant compounds, and VOC emissions. The decrease in growth parameters and lawsone content in the leaves was proportional to the salt level and the consequent reduction of photosynthetic CO_2_ uptake due to the partial stomatal closure. Overall, the preservation of photosynthetic apparatus from irreversible damage revealed a combination of biochemical and physiological adaptation responses to the salt stress, notwithstanding the lower production of economically important metabolites such as lawsone.

## Figures and Tables

**Table 1:** The influence of salt treatments (0, 75, and 150 mM NaCl) on leaf area and leaf number per plant determined during the growth of *Lawsonia inermis*. Relative water content (RWC, %) was determined at the end of treatment. Values are means ± Standard Error (SE) (*n* = 9). Different letters indicate statistically significant differences with Fisher’s probable least-squares difference test (*p* ≤ 0.05).

	Control 0 mM	75 mM NaCl	150 mM NaCl
Leaf number (0 DAT)	11.1 ± 0.69 ^b^	9.5 ± 1.4 ^a^	9.5 ± 0.69 ^a^
Leaf number (3 DAT)	11.25 ± 0.65 ^b^	10.75 ± 0.53 ^ab^	10 ± 0.38 ^a^
Leaf number (10 DAT)	14.62 ± 0.65 ^ab^	16.62 ± 1.18 ^b^	13.87 ± 0.67 ^a^
Leaf number (20 DAT)	21.5 ± 1.46 ^b^	19.87 ± 1.43 ^ab^	15 ± 0.54 ^a^
Leaf area (10 DAT, cm^2^ plant)	3.272 ± 0.446 ^b^	2.906 ± 0.22 ^ab^	2.089 ± 0.259 ^a^
Leaf area (20 DAT, cm^2^ plant)	4.845 ± 0.616 ^b^	4.622 ± 0. 203 ^b^	2.744 ± 0.163 ^a^
RWC (20 DAT, %)	80.29 ± 2.84 ^b^	71.63 ± 2.61 ^a^	74.35 ± 2.17 ^ab^

**Table 2:** Determination of pigments, secondary metabolites, and radical scavenger activity in the *Lawsonia inermis* leaves after treatment with different NaCl concentrations (0, 75, and 150 mM). Data are presented as means ± SE (*n* = 3). Different letters indicate statistically significant differences with Fisher’s probable least squares difference test (*p* ≤ 0.05). Abbreviations: GAE—gallic acid equivalents; CE—catechin equivalents; ME—malvin hloride equivalents.

	Control 0 mM	75 mM NaCl	150 mM NaCl
Chlorophyll *a* (Chla, µg cm^−2^ FW)	12.47 ± 0.56 ^a^	15.68 ± 1.17 ^a^	20.95 ± 0.28 ^b^
Chlorophyll *b* (Chlb, µg cm^−2^ FW)	3.70 ± 0.43 ^a^	5.22 ± 0.36 ^ab^	6.7 ± 0.52 ^b^
Total chlorophyll (Tchl, µg cm^−2^ FW)	16.17 ± 0.89 ^a^	20.9 ± 1.46 ^b^	27.65 ± 0.39 ^c^
Ratio Chla/Chlb	3.45 ± 0.38 ^b^	3.01 ± 0.16 ^a^	3.17 ± 0.26 ^ab^
Total carotenoids (Tcar, µg cm^−2^ FW)	5.2 ± 0.22 ^a^	5.86 ± 0.5 ^a^	7.4 ± 0.33 ^b^
Ratio Tcar/Tchl	0.32 ± 0.009 ^b^	0.28 ± 0.004 ^a^	0.267 ± 0.008 ^a^
Proline (mg g^−1^ FW)	70.16 ± 7.78 ^a^	76.08 ± 3.68 ^ab^	91.3 ± 4.67 ^b^
Total polyphenols (TP, mg GAE g^−1^ FW)	839.77 ± 55.598 ^a^	755 ± 27.7 ^a^	795.49 ± 26.86 ^a^
Total flavonoids (TF, mg CE g^−1^ FW)	375.82 ± 27.92 ^a^	448.48 ± 30.12 ^ab^	551.52 ± 65.28 ^b^
Total anthocyanins (mg ME g^−1^ FW)	29.35 ± 0.42 ^a^	34.53 ± 1.21 ^a^	48.96 ± 5.52 ^b^
Radical scavenging assay (DPPH-IC_50_ mg mL^−1^)	24.51 ± 3.09 ^b^	19.72 ± 0.05 ^ab^	16.072 ± 0.22 ^a^
Antioxidant activity-FRAP assay (mmol Fe^2+^ g^−1^ FW)	44.66 ± 1.71 ^a^	52.67 ± 1.74 ^ab^	58.66 ± 4.30 ^b^

**Table 3:** Results of the lawsone extraction process and its quantitative analyses in *Lawsonia inermis* aerial parts subjected to control (0 NaCl) and saline treatments (75 and 150 mM NaCl). Data are presented as means ± SD (*n* = 3). Different letters indicate statistically significant differences with Fisher’s probable least-squares difference test (*p* ≤ 0.05).

Treatment	Starting Dried Aerial Parts (g)	Yield of the Extraction Process (g)	Lawsone (mg g^−1^FW)
0 NaCl	1.0	0.1450	5.90 ± 0.1 ^b^
75 mM	1.0	0.0200	1.38 ± 0.2 ^a^
150 mM	1.0	0.0200	1.16 ± 0.1 ^a^

**Table 4:** Chemical composition of the volatile organic compounds (VOCs) from fresh leaves of *Lawsonia inermis* plants grown with variable salinity concentrations. HeadSpace–Solid Phase MicroExtraction (HS-SPME) was performed on Gas Chromatography–Mass Spectrometry (GC–MS) with DB-5 capillary column. Data represent mean values of relative percentage (*n* = 3, ± SD).

			Relative Percentage		
Compounds	LRI ^§^	Chemical Class	Control	75 mM NaCl	150 mM NaCl
(*E*)-3-Hexen-1-ol *	868	ALC	27.0 ± 2.33	6.2 ± 0.64	8.3 ± 0.64
1-Hexanol *	875	ALC	3.9 ± 0.17	2.1 ± 0.16	3.9 ± 0.17
Santolina triene	911	MH	0.4 ± 0.16	1.3 ± 0.14	0.5 ± 0.05
α-Fanchene	951	MH	*–*	0.4 ± 0.05	0.3 ± 0.15
6-Methyl-5-hepten-2-one	978	KET	0.5 ± 0.24	1.0 ± 0.10	0.3 ± 0.01
myrcene	993	MH	0.5 ± 0.14	1.7 ± 0.44	0.4 ± 0.05
6-Methyl-5-hepten-2-ol	995	ALC	0.6 ± 0.05	1.3 ± 0.14	0.2 ± 0.03
*m*-Mentha-1(7),8-diene	1001	MH	*–*	0.7 ± 0.07	*–*
*p*-Mentha-1(7),8-diene	1004	MH	*–*	1.2 ± 0.13	*–*
(*Z*)-3-Hexenyl acetate *	1008	EST	7.9 ± 0.58	3.4 ± 0.34	3.3 ± 0.34
*n*-Hexyl acetate *	1013	EST	*–*	*–*	0.2 ± 0.02
*o*-Cymene	1026	MH	*–*	0.3 ± 0.02	*–*
3-Ethyl-1-hexanol *	1032	ALC	1.9 ± 0.24	*–*	*–*
1,8-Cineole	1036	OM		3.5 ± 0.37	1.3 ± 0.16
(*Z*)-β-Ocimene	1042	MH	2.3 ± 0.32	1.2 ± 0.70	*–*
(*E*)-β-Ocimene	1053	MH	0.4 ± 0.09	0.5 ± 0.05	0.5 ± 0.29
γ-Terpinene	1062	MH	0.9 ± 0.40	*–*	*–*
*cis*-Sabinene hydrate	1072	OM	*–*	0.6 ± 0.01	*–*
*trans*-Arbusculone	1077	OM	*–*	0.2 ± 0.03	*–*
Fenchone	1090	OM	*–*	0.3 ± 0.03	*–*
Linalool	1102	OM	10.8 ± 0.63	11.9 ± 1.22	6.4 ± 0.57
*n*-Nonanal *	1104	ALD	5.5 ± 0.33	6.0 ± 0.62	1.8 ± 0.19
β-Thujone	1120	OM	0.3 ± 0.01	1.0 ± 0.07	0.1 ± 0.00
2-Ethylhexanoic acid	1123	EST	*–*	*–*	1.7 ± 0.22
Camphor	1148	OM	1.4 ± 0.21	2.0 ± 0.21	1.5 ± 0.50
Isoborneol	1160	OM	*–*	0.2 ± 0.10	*–*
Borneol	1169	OM	0.9 ± 0.31	1.0 ± 0.10	0.8 ± 0.07
Neo-*iso*-isopulegol	1171	OM	1.0 ± 0.06	1.0 ± 0.02	0.6 ± 0.15
4-Terpineol	1180	OM	0.6 ± 0.46	0.8 ± 0.08	0.5 ± 0.14
α-Terpineol	1192	OM	2.3 ± 0.25	2.9 ± 0.31	1.7 ± 0.26
*n*-Decanal	1206	ALD	4.7 ± 0.80	6.6 ± 0.69	3.3 ± 0.13
(*Z*)-3-Hexenyl 3-methylbutanoate *	1233	EST	*–*	*–*	0.6 ± 0.14
Hexyl 3-methylbutanoate *	1242	EST	*–*	*–*	1.8 ± 0.26
(*Z*)-3-Hexenyl isovalerate *	1243	EST	*–*	*–*	20.9 ± 2.33
Linalyl acetate	1260	OM	1.9 ± 0.17	1.8 ± 0.18	1.4 ± 0.24
Citronellyl formate	1280	OM	*–*	0.8 ± 0.08	1.1 ± 0.17
Lavandulyl acetate	1289	OM	2.1 ± 0.13	2.6 ± 0.26	1.9 ± 0.37
*n*-Tridecane	1300	ALK	*–*	0.5 ± 0.06	2.1 ± 0.59
Undecanal	1305	ALD	*–*	0.8 ± 0.09	*–*
Neryl acetate	1368	OM	0.9 ± 0.09	0.9 ± 0.09	0.6 ± 0.08
Geranyl acetate	1386	OM	2.6 ± 0.48	2.2 ± 0.23	1.4 ± 0.42
*n*-Tetradecane	1400	ALK	*–*	*–*	1.0 ± 0.05
Dodecanal	1409	ALD	*–*	1.0 ± 0.10	1.1 ± 0.10
*cis*-α-Bergamotene	1417	SH	*–*	*–*	0.6 ± 0.06
β-Caryophyllene	1418	SH	*–*	*–*	0.6 ± 0.19
(*E)*-Geranylacetone	1455	AC	9.8 ± 1.67	6.2 ± 0.64	1.2 ± 0.34
(*E*)-β-Farnesene	1460	SH	*–*	*–*	1.4 ± 0.18
*n*-Pentadecane	1500	ALK	0.6 ± 0.08	1.4 ± 0.15	7.1 ± 0.96
β-Bisabolene	1509	SH	*–*	*–*	0.3 ± 0.00
*trans*-γ-Cadinene	1513	SH	*–*	*–*	0.6 ± 0.14
Tetradecanal	1612	ALD	*–*	*–*	0.5 ± 0.09
τ-Cadinol	1642	OS	0.9 ± 0.31	4.9 ± 0.50	2.5 ± 1.26
α-Cadinol	1655	OS	*–*	0.5 ± 0.05	*–*
Octyl ether	1677	ETR	0.8 ± 0.10	1.5 ± 0.16	0.4 ± 0.18
Epi-α-bisabolol	1685	OS	*–*	3.1 ± 0.32	1.6 ± 0.23
*n*-Heptadecane	1700	ALK	*–*	0.7 ± 0.07	0.4 ± 0.22
(*E*)-Conipheryl alcohol	1727	PP	1.4 ± 0.08	4.3 ± 1.68	2.5 ± 0.25
*n*-Octadecane	1800	ALK	*–*	*–*	0.3 ± 0.01
β-Chenopodiol	1810	OS	*–*	0.6 ± 0.27	*–*
Octyl salicylate	1816	PP	*–*	*–*	1.1 ± 0.38
*Iso*propyl tetradecanoate	1824	EST	*–*	*–*	0.6 ± 0.08
(*E,E*)-Farnesyl acetate	1843	OS	2.2 ± 0.57	*–*	1.5 ± 0.82
Methyl3,5-di-tert-butyl-4-hydroxybenzoate	1859	EST	2.1 ± 0.19	5.2 ± 0.34	3.5 ± 0.55
*n*-Nonadecane	1899	ALK	*–*	0.4 ± 0.18	0.2 ± 0.00
*n*-Eicosane	2000	ALK	0.2 ± 0.01	*–*	*–*
*Iso*propyl hexadecanoate	2027	EST	*–*	0.5 ± 0.14	*–*
**Class of Compounds**			**Control**	**75 mM**	**150 mM**
Monoterpene hydrocarbons			4.5 ± 1.11	7.3 ± 1.38	1.7 ± 0.53
Oxygenated monoterpenes			24.8 ± 2.79	33.7 ± 3.41	19.3 ± 3.13
Sesquiterpene hydrocarbons			0.0 ± 0.00	0.0 ± 0.00	3.5 ± 0.57
Oxygenated sesquiterpenes			3.1 ± 0.88	9.1 ± 1.14	5.6 ± 2.31
Total terpenes			32.4 ± 4.78	50.1 ± 5.88	30.1 ± 6.54
Phenylpropanoids			1.4 ± 0.08	4.3 ± 1.68	3.6 ± 0.63
Apocarotenoides			9.8 ± 1.67	6.2 ± 0.64	1.2 ± 0.34
Alcohol			33.4 ± 2.79	9.6 ± 0.94	12.4 ± 0.85
Aldehydes			10.2 ± 1.13	14.4 ± 1.50	6.7 ± 0.51
Alkane			0.8 ± 0.09	3.0 ± 0.57	11.2 ± 1.83
Ester			10.0 ± 0.78	9.1 ± 0.69	32.6 ± 3.94
Ether + ketone			1.3 ± 0.34	2.5 ± 0.26	0.7 ± 0.19
Non-terpene derivatives			55.5 ± 5.13	38,6 ± 3.96	63.5 ± 7.33
**Total Identified**			**99.3** ± 0.66	**99.2** ± 0.31	**98.4** ± 0.95

* Compounds present with abundance ≥0.1%; ^§^ LRI: linear retention indices on DB-5 column; *: green leaf volatile compounds (GLV).

**Table 5:** Effect of salinity on volatile organic compounds (VOCs) according to the one-way PERMANOVA analysis.

	*F*	*p*	Significant Pair-Wise Comparisons at *p* < 0.05
Salinity	138.7	0.0003	Control versus 75 mM NaCl (*p* = 0.0291) Control versus 150 mM NaCl (*p* = 0.0263) 150 mM NaCl versus 75 mM NaCl (*p* = 0.093)

**Table 6:** List of compounds responsible for dissimilarity in *L. inermis* spontaneous emission according to the similarity percentage (SIMPER) analysis.

Compounds	Contribution %	Cumulative %	Control	75 mM NaCl	150 mM NaCl	Significant Pair-Wise Comparisons at *p* < 0.05 *
(*Z*)-3-Hexenyl isovalerate *	39.86	39.86	0.0	0.0	20.9	1 vs. 3, 2 vs. 3
(*E*)-3-Hexen-1-ol *	35.99	75.85	27.0	6.2	8.3	1 vs. 2, 1 vs. 3
(*E*)-Geranyl acetone	5.28	81.13	9.8	6.2	1.2	1 vs. 2, 1 vs. 3, 2 vs. 3
n-Pentadecane	3.47	84.60	0.6	1.4	7.1	1 vs. 3, 2 vs. 3
Linalool	2.45	87.05	10.8	11.9	6.4	1 vs. 3, 2 vs. 3
(*Z*)-3-Hexenyl acetate *	1.91	88.96	7.9	3.4	3.3	1 vs. 2, 1 vs. 3
n-Nonanal *	1.46	90.42	5.5	6.0	1.8	1 vs. 3, 2 vs. 3
τ-Cadinol	1.23	91.65	0.9	4.9	2.5	1 vs. 2, 1 vs. 3, 2 vs. 3
1,8-Cineole	0.86	92.51	0.0	3.5	1.3	1 vs. 2, 1 vs. 3, 2 vs. 3
n-Decanal	0.82	93.33	4.7	6.6	3.3	1 vs. 2, 1 vs. 3, 2 vs. 3
(*E*)-Conipheryl alcohol	0.78	94.11	1.4	4.3	2.5	1 vs. 2, 2 vs. 3
Methyl 3,5-di-tert-butyl-4-hydroxybenzoate	0.68	94.79	2.1	5.2	3.5	1 vs. 2, 1 vs. 3, 2 vs. 3
Epi-α-bisabolol	0.66	95.45	0.0	3.1	1.6	1 vs. 2, 1 vs. 3, 2 vs. 3

* 1: Control; 2: 75 mM NaCl; 3: 150 mM NaCl.
